# Genomic characterization of *mcr-1.1*-positive *Salmonella* Kentucky ST198 from a captive Asian houbara bustard reveals links to broiler poultry in the United Arab Emirates

**DOI:** 10.3389/fvets.2026.1831875

**Published:** 2026-05-12

**Authors:** Mar Carrasco Muñoz, Ana Pérez de Vargas, Mezna Alhebshi, Xander Velkeneers, Loïc Lesobre, Glindya Bhagya Lakshmi, Fatma A. Mohamed, Mohammed Elbediwi, Ihab Habib

**Affiliations:** 1 RENECO International Wildlife Consultants Ltd., Abu Dhabi, United Arab Emirates; 2 OIKOS Genomics, Abu Dhabi, United Arab Emirates; 3 Veterinary Public Health Research Laboratory, Department of Veterinary Medicine, College of Agriculture and Veterinary Medicine, United Arab Emirates University, Al Ain, United Arab Emirates; 4 Department of Microbiology and Immunology, Faculty of Pharmacy, Zagazig University, Zagazig, Egypt; 5 Department of Medical Microbiology and Immunology, University of Pécs Medical School, Pécs, Hungary; 6 University Institute for Medical Microbiology and Virology, Carl von Ossietzky University Oldenburg, Oldenburg, Germany; 7 Agriculture Research Center, Animal Health Research Institute, Cairo, Egypt

**Keywords:** Houbara bustard, mcr-1.1, One Health, *Salmonella* Kentucky, United Arab Emirates, whole-genome sequencing

## Abstract

The Asian houbara bustard (*Chlamydotis macqueenii*) is a vulnerable species supported by conservation breeding programs internationally, as well as in the United Arab Emirates (UAE). Infectious diseases caused by antimicrobial-resistant pathogens represent an emerging threat to captive populations. This study aimed to characterize the genomic features, antimicrobial resistance (AMR) determinants, and phylogenomic relatedness of an *mcr-1.1*-positive multidrug-resistant *Salmonella* Kentucky ST198 isolate recovered from a captive Asian houbara bustard in the United Arab Emirates. Post-mortem bacteriological culture of yolk sac samples yielded *S*. Kentucky isolate FAZ18016, which underwent antimicrobial susceptibility testing and long-read whole-genome sequencing. The isolate exhibited phenotypic multidrug resistance to 17 antimicrobials, supported by 21 resistance genomic determinants including *bla*_TEM − 1_, *mph(A), tet(A), floR, sul1/sul3*, and *mcr*-1.1. The colistin minimum inhibitory concentration was 8 mg/L. The *mcr*-1.1 gene was located on an IncHI2 plasmid co-harboring *tet(A), terC*, and multiple insertion sequences, sharing >99% nucleotide identity with plasmids described in *Klebsiella pneumoniae* and *Escherichia coli*. A total of 156 virulence-associated genes were identified, including complete Salmonella Pathogenicity Island 1 and 2, type III secretion systems and type VI secretion system components. Core-genome single-nucleotide polymorphism analysis assigned FAZ18016 to sequence type (ST) 198 and revealed 31–166 Single nucleotide polymorphism (SNP) differences from previously characterized UAE broiler isolates, with the closest related strains differing by 31–33 SNPs, consistent with relatively recent common ancestry. These findings indicate potential spillover of a high-risk, *mcr*-1.1–carrying *S*. Kentucky ST198 clone from poultry into captive wildlife and highlight the need for integrated genomic AMR surveillance and strengthened biosecurity at the wildlife–poultry interface within a One Health framework.

## 1 Introduction

The Asian houbara bustard (*Chlamydotis macqueenii*) inhabits semi-arid regions of Central Asia and the Middle East (1). Overhunting and habitat loss caused severe declines in wild houbara populations during the late 20th century leaving the species to be listed as Vulnerable by the International Union for Conservation of Nature (IUCN) (1). Given its high cultural and economic value for falconry in the United Arab Emirates (UAE), Abu Dhabi authorities have established a conservation program combining *in-situ* and *ex-situ* measures (2). While *ex-situ* conservation efforts have achieved notable success in reinforcing natural populations (3), conservation breeding projects are challenging and require extremely effective biosecurity measures (4). Captive populations are vulnerable to outbreaks of viral, bacterial, and parasitic diseases, which can cause significant mortality (4). Avian influenza virus, *Avipoxvirus*, Newcastle disease virus (*Avian orthoavulavirus 1), Escherichia coli*, and *Salmonella* spp. are among the infectious agents that cause illness and can lead to death in captive houbara (5–7).

*Salmonella* spp. are globally important zoonotic pathogens of wild and domestic birds, associated with both subclinical carriage and large mortality events, and can act as reservoirs for transmission to humans and livestock (8, 9). In houbara and other bustard species, opportunistic and zoonotic bacteria are frequently isolated from cloacal swabs and from birds examined at necropsy (10, 11). A recent study in Pakistan reported high prevalence of *Salmonella* spp. in captive Asian houbara bustards, with many isolates showing multidrug resistance (MDR) (10). More broadly, wild and migratory birds are increasingly recognized as sentinels and potential disseminators of extended-spectrum β-lactamase (ESBL)–producing and other MDR Enterobacterales along flyways and across continents (12).

Among *Salmonella* serovars, *S. enterica* serovar Kentucky ST198 has emerged as a high-risk lineage with extensive antimicrobial resistance (AMR) to fluoroquinolones and other antimicrobials (13). The serovar Kentucky is now widely distributed along poultry value chains and in travel-associated human infections and is considered an important transboundary AMR threat (14, 15). In the UAE, recent work has documented MDR *Salmonella*—including ESBL and *mcr*-positive strains—along broiler production, slaughter and retail chilled chicken meat chains, as well as in imported poultry products (16–18). Despite these concerns, information on high-risk *Salmonella* lineages at the interface between intensive poultry production and threatened wildlife in the Middle East remains scarce. In this context, the limited baseline data on *Salmonella* infections in houbara bustards highlight an important knowledge gap and an opportunity to generate evidence that can inform and strengthen effective preventive health strategies in breeding centers.

In the present work, we describe a septicemic yolk sac infection associated with colistin-resistant *S. enterica* serovar Kentucky ST198 carrying *mcr*-1.1 in a captive Asian houbara bustard chick. The objective of this study was not only to describe a clinical infection in a captive Asian houbara bustard, but also to perform an in-depth genomic characterization of the recovered *Salmonella* strain, including its resistome, virulome, and *mcr-1.1*-bearing plasmid architecture, and to compare it phylogenomically with poultry-associated isolates from the UAE to investigate possible One Health linkages between captive wildlife and broiler reservoirs.

## 2 Materials and methods

### 2.1 Study setting and clinical case

The investigation was conducted at the National Avian Research Center (NARC) in the Emirate of Abu Dhabi, UAE (https://houbarafund.gov.ae/). A captive Asian houbara chick hatched in December 2024 following artificial insemination of a captive female and standard incubation in a dedicated hatchery. The incubation period (23 days) and hatch parameters were within expected ranges for the species under captive conditions (22 ± 1 days) (19). Routine post-hatch evaluation on day 1 showed a closed umbilicus, normal activity, and good plumage condition. Growth was initially normal but deviated from the internal growth standard for captive-bred chicks from day 6 post-hatch, when reduced weight gain and mild lethargy were noted. On day 9, clinical examination revealed distension of the coelomic cavity with a firm, non-fluctuant mass on palpation, consistent with a retained, non-resorbed yolk sac and suspected yolk sacculitis. Supportive therapy was instituted (0.9% sodium chloride and lactated Ringer's solution, 10% glucose and multivitamin supplementation), together with empirical enrofloxacin administered orally. The choice of enrofloxacin was based on contemporary sensitivity testing from houbara chicks of similar age presenting with comparable signs in the same facility.

Despite supportive care, the chick failed to improve. Given the poor prognosis and strong suspicion of bacterial yolk sac infection, euthanasia via intravenous injection of T61 (MSD, 200 mg Embutramide, 50 mg Mebezonium Iodide and 5 mg Tetracaine Hydrochloride) was elected on welfare grounds at 13 days of age (20). A complete necropsy was performed immediately after death, revealing a non-resorbed yolk sac containing firm, inspissated material, and bilaterally enlarged kidneys. This case was investigated as an isolated individual presentation, with no evidence of a flock-level outbreak at the time of examination.

### 2.2 Bacteriological identification

At necropsy, the yolk sac content was aseptically sampled using an Amies transport swab (Copan Diagnostics, Italy). The specimen was inoculated onto non-selective and differential media, including Hektoen enteric agar and mannitol salt agar (HiMedia, India), and incubated aerobically at 37 °C with plate readings at 24 and 48 h. For selective enrichment of *Salmonella*, an aliquot of the swab was inoculated into Müller–Kauffmann tetrathionate broth (MKTT) and incubated for 24 h at 42 °C prior to subculture onto Hektoen enteric agar (Oxoid, UK), following standard methods for isolation from avian tissues (10). Presumptive bacterial colonies were purified and characterized by colony morphology and standard biochemical testing, including Analytical Profile Index (API) 20E test strips (bioMérieux, France). Species-level confirmation was obtained using matrix-assisted laser desorption ionization–time-of-flight mass spectrometry (MALDI-TOF MS; Autobio Diagnostics, China) (17).

Serogrouping of *Salmonella* was performed by slide agglutination using commercial polyvalent and monovalent antisera (Bio-Rad Laboratories, USA) according to the Kauffmann–White–Le Minor scheme, and the isolate was stored at −80 C in brain–heart infusion broth with 10% glycerol for downstream genomic and phenotypic analyses.

### 2.3 Antimicrobial susceptibility testing

Phenotypic antimicrobial susceptibility testing (AST) for the *S*. Kentucky isolate FAZ18016 was undertaken using the Vitek 2 Compact automated system. MICs were determined with the GN AST-GN96 card following the manufacturer's instructions (bioMérieux, France). The Vitek 2 panel included 17 agents commonly used in veterinary medicine and poultry practice: ampicillin, amoxicillin–clavulanic acid, ticarcillin–clavulanic acid, cefalexin, cefalotin, cefoperazone, ceftiofur, cefquinome, imipenem, gentamicin, neomycin, flumequine, enrofloxacin, marbofloxacin, tetracycline, florfenicol and trimethoprim–sulfamethoxazole.

Colistin susceptibility (and other antimicrobials such as azithromycin and chloramphenicol) was assessed separately by broth microdilution using Sensititre™ EUVSEC3 plates (Thermo Fisher Scientific, UK) in accordance with the manufacturer's instructions. Isolates with Colistin MICs > 2 mg/L were classified as non-wild type, consistent with acquisition of a resistance mechanism (16).

### 2.4 Genomic DNA extraction and long-read sequencing

Genomic DNA was extracted from the *Salmonella* isolate recovered from the yolk sac (FAZ18016) using the DNeasy Blood & Tissue Kit (Qiagen, Hilden, Germany) according to the manufacturer's protocol. DNA concentration and purity were assessed by spectrophotometry (NanoDrop™, Thermo Fisher Scientific, Waltham, MA, USA) and fluorometric quantification (Quantus™, Promega, Madison, WI, USA) (16). For *Salmonella* FAZ18016 isolated from the deceased houbara, Oxford Nanopore long-read sequencing was performed by OIKOS Genomics (Abu Dhabi, UAE). Libraries were prepared using the Rapid PCR Barcoding Kit (SQK-RPB114.24; Oxford Nanopore Technologies, Oxford, UK) and sequenced on a MinION flow cell (FLO-MIN114; Oxford Nanopore Technologies, Oxford, UK) with live super-accurate (SUP) simplex basecalling and simultaneous demultiplexing in MinKNOW (v25.03.7), following the manufacturer's instructions (Oxford Nanopore Technologies, UK).

To contextualize FAZ18016 within the local collection of the same serovar (identified as *S*. Kentucky) and to explore potential lineage connectivity across the wildlife–broiler interface, we performed whole-genome single-nucleotide polymorphism (SNP) analysis on the Solu Genomics platform (21). The poultry-derived *Salmonella enterica* serovar Kentucky isolates included for comparison with the houbara-derived isolate FAZ18016 were annotated with relevant epidemiological and genomic metadata, including isolate identifier, sequence type, geographical origin, sample source, year of isolation, NCBI BioSample accession number, and antimicrobial resistance genotype. This information is provided in Table 1 to document the strain set used for comparative analysis. The nine genomes included (Table 1) represent the full set of currently published *S*. Kentucky genomes from the UAE available to us at the time of analysis (16–18).

**Table 1:** Metadata and antimicrobial resistance genotypes of poultry-derived *Salmonella enterica* serovar Kentucky isolates included in the comparative genomic analysis with the houbara bustard isolate FAZ18016.

Isolates	Sequence type	Origin	Source	Year of isolation	NCBI biosample	Antimicrobial resistance genotype
SAL 5	ST198	United Arab Emirates	Retail chicken meat (company E)	2021	SAMN52951201	*aac*(3)−*Id, aadA*7, *aph*(6)−*Id, bla*_TEM − 1_*, gyrA_D87Y, gyrA_S83F, parC_S80I, qacEdelta1, sul1, tet(A)*
SAL 26	ST198	United Arab Emirates	Retail chicken meat (company C)	2021	SAMN52951200	*bla*_TEM − 1_ *, gyrA_D87Y, gyrA_S83F, parC_S80I*
SAL 33	ST198	United Arab Emirates	Retail chicken meat (company C)	2022	SAMN52951199	*aac*(3)−*Id, aadA*7, *aph*(6)−*Id, bla*_TEM − 1_*, gyrA_D87Y, gyrA_S83F, parC_S80I, qacEdelta1, sul1, tet(A)*
SAL 57	ST198	United Arab Emirates	Retail chicken meat (company G)	2022	SAMN52951196	*aac*(3)−*Id, aadA*7, *bla*_TEM − 1_*, gyrA_D87Y, gyrA_S83F, parC_S80I, qacEdelta1, sul1, tet(A)*
SAL 58	ST198	United Arab Emirates	Retail chicken meat (company G)	2022	SAMN52951195	*aac*(3)−*Id, aadA*7, *bla*_TEM − 1_*, floR, fosA4, gyrA_D87Y, gyrA_S83F, parC_S80I, qacEdelta1, sul1, tet(A), tet(X4)*
SAL 111	ST198	Ukraine	Reali imported frozen chicken meat	2023	SAMN52951197	*qnrB19*
SAL 120	ST152	Oman	Reali imported frozen chicken meat	2023	SAMN52951198	*aph(3″)-Ib, aph(6)-Id, tet(B)*
SAL 178	ST198	United Arab Emirates	Abattoir broiler carcass (company C)	2024	SAMN52951194	*aac*(3)−*Id, aadA*7, *aph*(6)−*Id, bla*_TEM − 1_*, gyrA_D87Y, gyrA_S83F, parC_S80I, qacEdelta1, sul1, tet(A)*
SAL 186	ST198	United Arab Emirates	Abattoir broiler carcass (company C)	2024	SAMN52951193	*aac*(3)−*Id, aadA*7, *aph*(6)−*Id, bla*_TEM − 1_*, gyrA_D87Y, gyrA_S83F, parC_S80I, qacEdelta1, sul1, tet(A)*

### 2.5 Genome *in silico* characterization and comparative phylogenomic

Basecalled FASTQ files for isolate FAZ18016 were uploaded to the Solu Genomics cloud platform (https://www.solugenomics.com) for bacterial genome analysis (21). Long reads were quality controlled using nanoq v0.10.0 (https://github.com/esteinig/nanoq), and pre-processed, assembled and polished with Dragonflye v1.2.1 (https://github.com/rpetit3/dragonflye). Validated workflows encompassed *in silico* serovar prediction, multilocus sequence typing (MLST), AMR gene and mutation detection, plasmid replicon typing, and virulence factor screening. Serovar assignment used SISTR (v1.1.3); sequence type was determined using the *S. enterica* Achtman 7-locus MLST scheme via PubMLST.v2.23.0 (https://github.com/tseemann/mlst). AMR determinants and relevant chromosomal point mutations were identified with AMRFinderPlus (v3.11.20). Plasmid incompatibility groups were inferred using MOB-suite (v3.1.9), and virulence-related loci were screened with Abricate (v1.0.1) against the Virulence Factor Database (VFDB) (https://www.mgc.ac.cn/VFs/). The VFDB version was last updated on June 3, 2025. All the former tools were integrated and run through the Solu Genomics cloud platform v1.0.656 (21; https://www.solugenomics.com/).

Phylogenetic relationships among *Salmonella* isolates were inferred using the Solu reference-based phylogeny pipeline v.1.2.7 (21). Briefly, genomes were aligned to a reference (strain LT2 (assembly GCF_000006945.2)) using Snippy v4.6.0 (https://github.com/tseemann/snippy), followed by generation of a core-genome alignment with snippy-core command from Snippy. Low-quality SNPs were filtered by excluding SNPs within 10 bp of another SNP and those within 15 bp of ambiguous bases. Pairwise SNP distances were calculated from the filtered alignment using snp-dists v0.8.2 (https://github.com/tseemann/snp-dists). Maximum-likelihood phylogenetic trees were inferred with IQ-TREE v2.3.6 (http://www.iqtree.org) after removal of constant sites using snp-sites v2.5.1 (https://github.com/sanger-pathogens/snp-sites), while accounting for removed constant bases to preserve branch lengths. Near-zero branch collapse was enabled to allow polytomies among highly similar isolates, and the final tree was formatted and midpoint-rooted using Biopython (https://github.com/biopython/biopython).

The genomes generated in this study have been deposited in the NCBI Sequence Read Archive (SRA) under BioProject accession number PRJNA1354649 (*Salmonella* ST198 in UAE Houbara-Broiler) and are publicly available.

### 2.6 Mobile genetic elements characterization and plasmid reconstruction

Integrative mobile genetic elements (iMGEs) and flanking regions were detected using the MobileElementFinder pipeline hosted by the Center for Genomic Epidemiology (https://cge.food.dtu.dk/services/MobileElementFinder/) (22). This tool combines sequence similarity searches against curated repositories of known MGEs with predictive modeling to detect composite transposons and other structural elements, drawing on data from ISfinder, the Transposon Registry and ICEberg (22).

Targeted plasmid analysis was undertaken after MobileElementFinder screening revealed co-occurrence of an IncHI2 plasmid replicon and the colistin resistance determinant *mcr*-1.1 on a single long contig in the draft assembly. To demarcate plasmid from chromosomal sequence, we applied a BLAST-based workflow using BLASTn against the NCBI plasmid sequence collection. A sliding-window strategy (overlapping windows along the contig) was used to identify regions showing continuous, high-identity mapping to curated IncHI2 plasmids while minimizing erroneous assignment of chromosomal sequence (23, 24). Circular alignment and visual comparison of the reconstructed plasmid region against selected reference plasmids from *Klebsiella pneumoniae* and *Escherichia coli* were generated using the BLAST Ring Image Generator (BRIG) v0.95 (21), and the *mcr*-1.1 gene environment was compared to previously published *mcr*-bearing IncHI2 plasmids (23, 24).

## 3 Results

### 3.1 Phenotypic and genotypic characterization of antimicrobial resistance

The *S. enterica* serovar Kentucky isolate FAZ18016 displayed an extensive MDR phenotype, with resistance or reduced susceptibility to 17 antimicrobials (Table 2). Phenotypically, the isolate was resistant to ampicillin, amoxicillin–clavulanic acid, and ticarcillin–clavulanic acid, and to first-generation cephalosporins (cefalexin, cefalotin, cefoperazone), while showing intermediate MICs to ceftiofur but remaining susceptible to cefquinome and imipenem (Table 2). It also exhibited resistance to azithromycin, gentamicin, neomycin, flumequine, enrofloxacin, marbofloxacin, tetracycline, florfenicol and trimethoprim–sulfamethoxazole (Table 2). The colistin MIC by broth microdilution was 8 mg/L (Table 2), classifying the isolate as colistin non-wild type.

**Table 2:** Phenotype–genotype correlation of antimicrobial resistance in *mcr-1*–positive *Salmonella enterica* serovar Kentucky FAZ18016 isolated from a captive houbara bustard (*Chlamydotis macqueenii*) in the United Arab Emirates.

Antimicrobial class/agent	Resistance gene(s) detected	Minimum inhibitory concentration (mg/L)	Phenotypic interpretation
β-lactams			
Ampicillin	*bla*_TEM − 1_	≥32	Resistant
Amoxicillin–clavulanic acid	*bla*_TEM − 1_	≥32	Resistant
Ticarcillin–clavulanic acid	*bla*_TEM − 1_	≥128	Resistant
Cefalexin	—	≥64	Resistant
Cefalotin	—	≥64	Resistant
Cefoperazone	—	≥64	Resistant
Ceftiofur	—	4	Intermediate
Cefquinome	—	≤ 0.5	Susceptible
Imipenem	—	≤ 0.25	Susceptible
Macrolides			
Azithromycin	*mph(A)*	≥64	Resistant^*^
Phenicols			
Chloramphenicol	*cmlA1*	≥64	Resistant^*^
Florfenicol	*floR*	≥32	Resistant
Aminoglycosides			
Gentamicin	*aac(3)-Id*	≥16	Resistant
Neomycin	*aph(3′)-Ia, aph(3″)-Ib, aph(6)-Id*	≥64	Resistant
Streptomycin	*aadA1, aadA2, aadA7, aph(6)-Id*	—	Not tested
Quinolones/Fluoroquinolones			
Flumequine	*gyrA_S83F, gyrA_D87Y, parC_S80I mutations*	≥32	Resistant
Enrofloxacin	*gyrA_S83F, gyrA_D87Y, parC_S80I mutations*	≥4	Resistant
Marbofloxacin	*gyrA_S83F, gyrA_D87Y, parC_S80I mutations*	≥4	Resistant
Polymyxins			
Colistin	*mcr-1.1*	8	Resistant^*^
**Tetracyclines**	*tet(A)*	≥16	Resistant
**Sulfonamides**	*sul1, sul3*	—	Not tested
**Trimethoprim/Sulfonamide combination**	*dfrA14, sul1, sul3*	≥32	Resistant
**Quaternary ammonium compounds/biocide tolerance**	*qacEΔ1, qacL*	—	Not applicable

^*^Results based on Sensititre™ EUVSEC3 plate panel, while the rest were based on results of Vitek2 GN AST-GN96 card panel.

The long-read quality and assembly indicators for the sequencing of isolate FAZ18016 are summarized in Table 3. Long-read sequencing yielded 196 × genome coverage with a read N50 of 3,737 bp. Assembly produced a 5.37 Mb draft genome (within expected range of 4.40 Mbp-5.90 Mbp) in 25 contigs, with an assembly N50 of 457 kbp, 100.0% completeness, and 0.4% contamination (Table 3). WGS identified 21 resistance determinants consistent with the observed MDR phenotype (Table 2). These included *bla*_TEM − 1_ (β-lactams), *mph(A)* (macrolides), *aac(3)-Id, aph(3*′*)-Ia, aph(3*″*)-Ib* and *aph(6)-Id* (aminoglycosides), chromosomal mutations in *gyrA* and *parC* (fluoroquinolone resistance), *tet(A)* (tetracycline), *floR* and *cmlA1* (phenicols), and *dfrA14* in conjunction with *sul1* and *sul3* (trimethoprim–sulfonamide resistance). The plasmid-mediated colistin resistance gene *mcr*-1.1 was detected, alongside biocide tolerance genes *qacE*Δ*1* and *qacL* (Table 2).

**Table 3:** Long-read sequencing and assembly quality metrics for *Salmonella enterica* serovar Kentucky isolate FAZ18016 isolated from a captive houbara bustard (*Chlamydotis macqueenii*) in the United Arab Emirates.

Category	Metric^*^	Value
Long-read sequencing quality	Read N50	3,737 bp
	Median read length	3,512 bp
	Q15 rate	80.7%
	Coverage	196 ×
	Domain classification	Bacteria
Assembly quality	Genome size	5.37 Mbp
	GC content	51.9%
	Assembly N50	457 kbp
	Contig count	25
	Completeness	100.0%
	Contamination	0.4%

^*^Assembly completeness and contamination were estimated using the Solu analysis pipeline (https://www.solugenomics.com) (21).

### 3.2 Phylogenetic context and relatedness to poultry isolates

**Figure 1:**
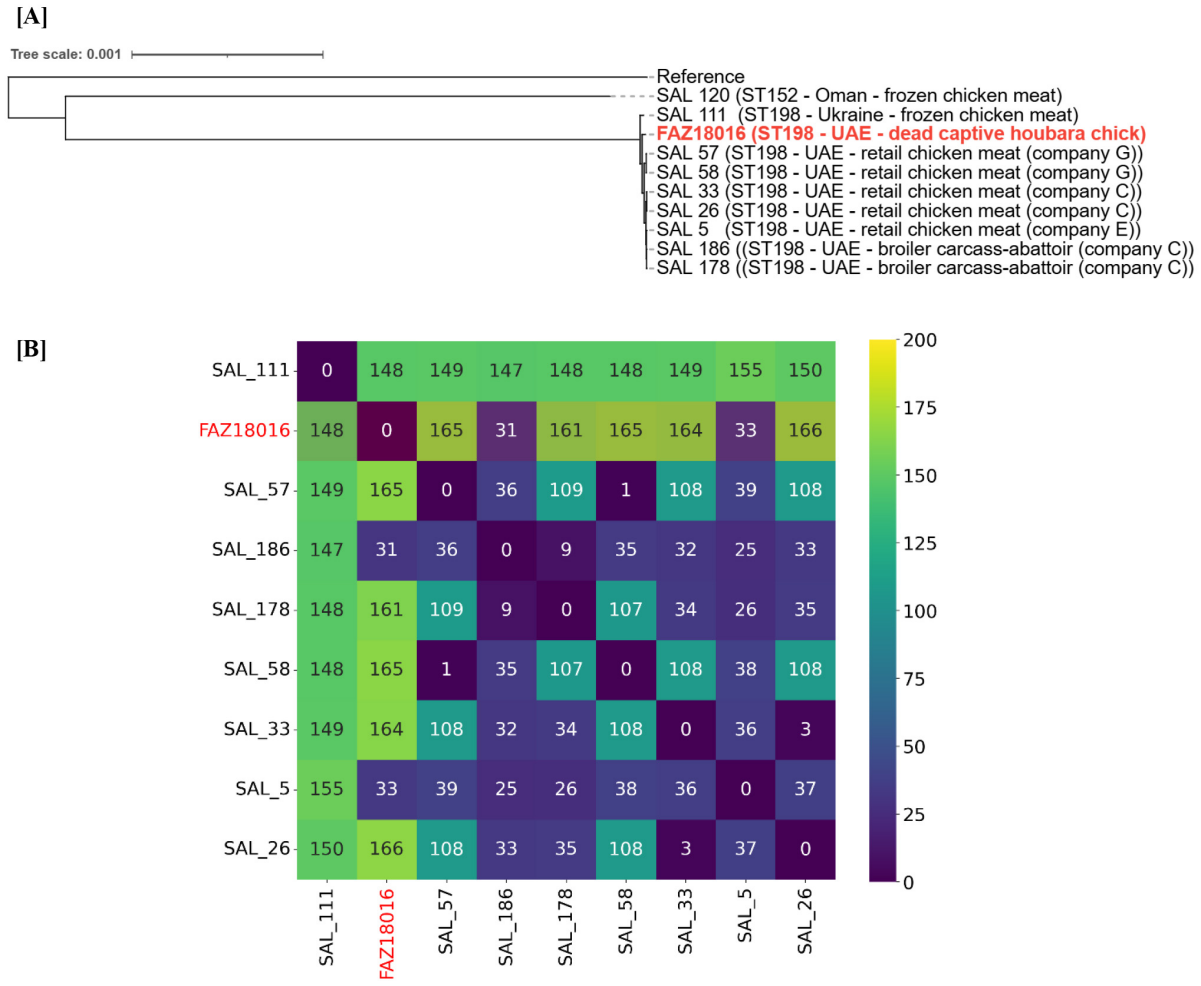
**(A)** Whole-genome single-nucleotide polymorphism (SNP)-based phylogeny ten *Salmonella enterica* serovar Kentucky isolates from the United Arab Emirates, including one isolate from a deceased captive Asian houbara bustard chick (FAZ18016), five from retail chicken meat (SAL 5, SAL 26, SAL 33, SAL 57, SAL 58), two from broiler carcasses sampled at abattoirs (SAL 178, SAL 186), and two from imported frozen retail chicken (SAL 111, SAL 120). The reference genome used was *Salmonella enterica* strain LT2 (assembly GCF_000006945.2). **(B)** Pairwise SNP (Single Nucleotide Polymorphism) differences among isolates belonging to Sequence Type (ST) 198 across houbara-broiler interface.

Whole-genome single-nucleotide polymorphism (SNP) based phylogenomic analysis placed FAZ18016 among nine *S*. Kentucky ST198 genomes previously characterized from poultry sources in the UAE, including isolates from retail chicken meat, broiler carcasses at abattoirs and imported frozen chicken (Figure 1A). Pairwise SNP distances among ST198 isolates across the houbara–broiler interface (including FAZ18016) ranged from 31 to 166 SNPs. The houbara isolate FAZ18016 showed the closest relatedness to two broiler-derived isolates from different producers (SAL_186 and SAL_5), differing by only 31–33 SNPs, retrospectively (Figure 1B).

### 3.3 Mobile genetic elements and virulence-associated loci

MobileElementFinder screening identified multiple MGEs associated with AMR and virulence loci (Table 4). The genome of the houbara isolate FAZ18016 harbored two plasmids of replicon types IncHI2 and IncI1 (Table 4). The *mcr*-1.1 gene co-localized with an IncHI2 replicon on contig00002, which also carried *tet(A)* and the tellurium resistance gene *terC*. This contig contained multiple insertion sequences and two composite transposons. Additional resistance genes were distributed on several other contigs: contig00016 harbored *aadA7, aac(3)-Id, sul1, qacE* and *bla*_*TEM*1−*B*_ alongside one insertion sequence and two composite transposons; contig00010 carried *cml, floR, mph(A), sul3, aadA1, aadA2b, aph(3*″*)-Ib, aph(6)-Id, dfrA14, qacL*, *bla*_TEM1 − B_ and *aph(3*′*)-Ia*, with two insertion sequences. Further loci included *aac(6*′*)-Iaa* on contig00001; *aph(6)-Id* with *tet(A)* on contig00018; and additional *tet(A)* on contig00019. Putative virulence or fitness genes were identified near MGEs, including *nlpI* and *iha* (contig00006), *shiB* (contig00004), and *cia* on an IncI1-bearing contig (contig00015). Several contigs (contig00003, contig00005, contig00007, contig00008) contributed additional insertion sequences and composite transposons without further annotated resistance genes (Table 4).

**Table 4:** Detection of mobile genetic elements associated antimicrobial resistance and virulence genes in genome contigs of *mcr-1*–positive *Salmonella enterica* serovar Kentucky FAZ18016 isolated from a captive houbara bustard (*Chlamydotis macqueenii*) in the United Arab Emirates.

Sequence	Plasmid	Resistance genes/predicted phenotype	Virulence genes/protein function	Other mobile elements	
				Insertion sequence	Composite transposon
contig00002	IncHI2	*mcr-1.1* (colistin), and *tet(A)* (doxycycline, tetracycline)	*terC* (tellurium ion resistance protein)	5	2
contig00016	—	*aadA7* (spectinomycin, streptomycin), *aac(3)-Id* (astromicin, fortimicin, gentamicin), *sul1* (sulfamethoxazole), *qacE* (ethidium bromide, cetylpyridinium chloride, benzylkonium chloride, chlorhexidine), and *bla_*TEM*−1*B*_* (ampicillin, cephalothin, amoxicillin, piperacillin, ticarcillin)	—	1	2
contig00010	—	*cml* (chloramphenicol), *floR* (florfenicol, chloramphenicol). *mph(A)* (spiramycin, azithromycin, erythromycin, telithromycin), *sul3* (sulfamethoxazole), *aadA1* and *aadA2b* (spectinomycin, streptomycin), *aph3(″)-Ib* and *aph(6)-Id* (streptomycin), *dfrA14* (trimethoprim), *qacL* (ethidium bromide, cetylpyridinium chloride, benzylkonium chloride, chlorhexidine), *bla_*TEM*−1*B*_*, and *aph(3′)-Ia* (neomycin, lividomycin, kanamycin, paromomycin, ribostamycin)	—	2	—
contig00001	—	*aac(6′)-Iaa* (amikacin, tobramycin)	—	—	—
contig00018	—	*aph(6)-Id* (streptomycin), and *tet(A)*	—	1	—
contig00019	—	*tet(A)*	—	—	—
contig00006	—	—	*nlpI* (lipoprotein NlpI precursor), and *iha* (Adherence protein)	—	—
contig00004	—	—	*shiB* (homologs of the Shigella flexneri SHI-2 pathogenicity island gene shiA)	—	—
contig00015	IncI1	—	*cia* (Colicin ia)	—	—
contig00003	—	—	—	2	1
contig00005	—	—	—	1	—
contig00007	—	—	—	2	—
contig00008	—	—	—	1	1

In-depth analysis of virulence-associated loci revealed 156 virulence factors in FAZ18016 (Supplementary Sheet 1). These included genes encoding both fimbrial and non-fimbrial adhesins, the macrophage-inducible factor *mig-14*, and multiple type III secretion system (T3SS) components located on Salmonella pathogenicity islands SPI-1 and SPI-2.

SPI-1 contained a full complement of invasion-associated genes (*hilA, hilC, hilD, invA–H, sipA–D, sicA, sptP, prgH–K*) and regulatory loci (*iagB, sicP, avrA*) required for epithelial invasion and early intracellular survival. SPI-2 was similarly intact, harboring the translocon and secretion apparatus (*ssaC–V, ssrA, ssrB*) and effector proteins *sseA–L, sifA, sifB, srfJ, pipB, pipB2, steA–D, steC, sseK1–2* and *sseJ*. Additional effectors outside the main pathogenicity islands included *sopA, sopB/sigD, sopD, sopE2* and *slrP*, which are known to modulate cytoskeletal rearrangements and inflammatory responses. Type VI secretion system (T6SS)–related genes (*vgrG, tssJ, tssK, tae4*) and toxin-associated loci (*tlde1*) were also present (Supplementary Sheet 1).

### 3.4 Genomic comparison of the *mcr*-1.1 gene environment and IncHI2 plasmid architecture

**Figure 2:**
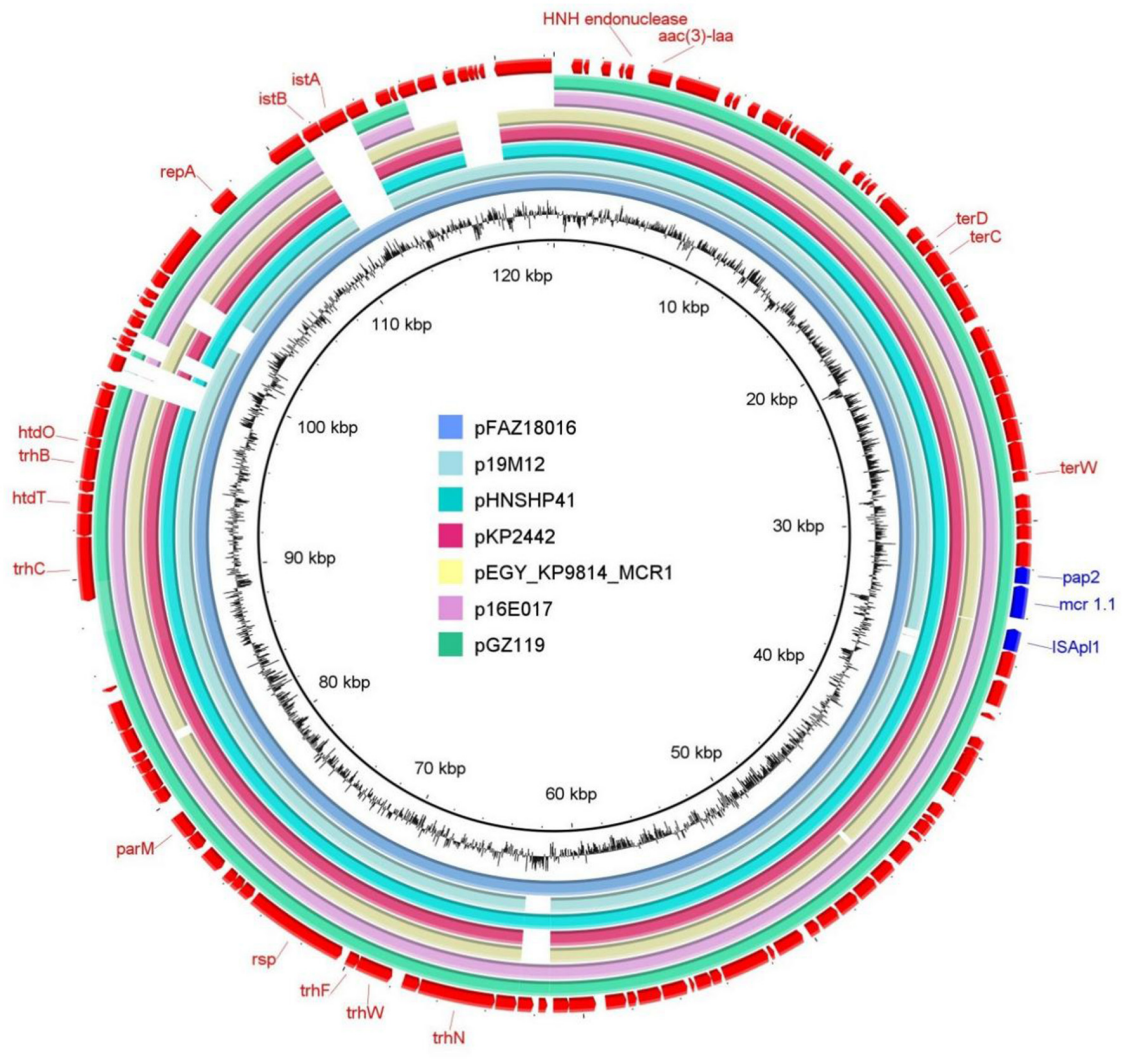
Circular comparison analysis visualized the reconstructed pFAZ18016 plasmid (innermost blue ring) harbored by *Salmonella enterica* serovar Kentucky isolated from a captive Asian houbara bustard in the United Arab Emirates. The pFAZ18016 is aligned against six related plasmids, demonstrating extensive genomic synteny across the IncHI2 family. Outer red arrows denote the annotated protein-coding genes and regulatory elements, while blue coloring highlights the *mcr*-1.1 (mobilizable colistin resistance gene) determinant and its associated genomic context including pap2 gene and ISApl1 insertion sequences.

Reconstruction of the *mcr*-1.1–bearing plasmid region revealed a long contig (contig00002; 515,226 bp) containing both the IncHI2 replicon and *mcr*-1.1 separated by approximately 73.5 kb. The *mcr*-1.1 locus mapped to positions 48,702–50,512 bp (reverse strand), while the IncHI2 replicon was located at positions 123,996–124,322 bp (Table 4, Figure 2).

Figure 2 shows that the reconstructed plasmid pFAZ18016 (122,399 bp) shared very high sequence similarity with previously characterized *mcr-*1-bearing plasmids from MDR clinical isolates. BLAST analysis demonstrated 99.97% nucleotide identity across 93% query coverage with *Klebsiella pneumoniae* strain KP2442 plasmid pKP2442_1c330 (KX434879.1; E-value = 0.0), and similarly high similarity to *K. pneumoniae* subsp. *pneumoniae* strain 9814 plasmid pEGY_KP9814_MCR1 (99.98% identity, 93% coverage; OQ215737.1). The plasmid also showed 100% identity to *E. coli* strain 19-M12 plasmid p19M12 over 95% coverage (KY689632.1), and 99.91-99.92% identity to *Salmonella enterica* plasmids over 91% coverage (PX833615.1 and PX759563.1) (Figure 2). Circular comparative analysis depicted in Figure 2 further confirmed conservation of the plasmid backbone, with the outer annotation track indicating predicted coding sequences and the *mcr*-1.1 region highlighted in blue together with its associated genetic context, including *pap2* (PAP2 family phosphatase gene) and ISApl2 (insertion sequence) elements. Collectively, these findings confirm the successful reconstruction of pFAZ18016 and place it within a closely related group of conjugative IncHI2 plasmids linked to colistin resistance (Figure 2).

## 4 Discussion

This study documents a septicemic infection with colistin-resistant *S. enterica* serovar Kentucky ST198 carrying *mcr*-1.1 in a captive Asian houbara bustard and demonstrates close phylogenetic relatedness to broiler-associated ST198 isolates from the UAE. To our knowledge, this is the first description of *mcr*-1.1–positive *S*. Kentucky in a bustard species; the present study provides genomic evidence of a One Health interface between conservation breeding systems and poultry-associated AMR reservoirs in the region.

The clinical course and lesions were consistent with septicemic yolk sac infection, a recognized cause of early chick mortality in poultry and other captive birds such as raptors (25). The isolation of MDR *S*. Kentucky as the predominant pathogen from the yolk sac, together with the extensive virulence repertoire detected by WGS, supports its primary etiological role. The presence of intact SPI-1 and SPI-2 T3SS, T6SS loci and multiple effectors associated with epithelial invasion and systemic spread indicates that this ST198 strain retains full pathogenic potential in non-galliform hosts and is not merely an adapted colonizer of the poultry gut (26).

High-level fluoroquinolone resistance is a hallmark of the globally disseminated ST198 clone and has been widely reported in poultry and human cases (13, 27). In FAZ18016, this MDR profile was further compounded by resistance determinants against β-lactams, macrolides, tetracyclines, phenicols and trimethoprim–sulfonamides, leaving only cefquinome and imipenem as fully active options *in vitro*. However, regulatory restrictions and stewardship considerations severely limit the use of such critically important antimicrobials in wildlife and veterinary settings in the UAE (28). Thus, the combination of broad MDR and high virulence substantially narrows therapeutic options for critically ill houbara and other captive wildlife, increasing the risk of fatal outcomes during outbreaks in conservation centers.

The plasmid architecture of FAZ18016—IncHI2 background carrying *mcr*-1.1, *tet(A), terC* and multiple insertion sequences, with near-identity to plasmids previously described in *E. coli* and *K. pneumoniae*—is consistent with broad-host-range IncHI2 vehicles that have played a major role in disseminating *mcr*-1 and other colistin resistance genes across human, animal and environmental compartments (29, 30). Once established in such plasmid backbones, *mcr* determinants can persist and circulate independently of current colistin use in a particular setting because selection is maintained by co-resident resistance or tolerance traits, including ones targeting disinfectants and metals (30).

In FAZ18016, co-location of *mcr*-1.1 with *tet(A), terC, qacE*Δ*1* and *qacL* suggests that exposure to tetracyclines, quaternary ammonium compounds (QACs) and tellurium or other metals could support plasmid persistence even in the context of policies aimed at reducing or banning colistin use (28). Studies from the region have already documented *mcr*-positive *E. coli* from poultry meat, dromedary camels and companion animals, often associated with IncI2, IncX4 and IncHI2 plasmids and co-resistance to other antimicrobials (31–33). At the global level, heavy metals and biocides have been recognized as important co-selectors of AMR, contributing to maintenance and spread of resistance genes in farm and environmental microbiomes (34).

Although the UAE has taken steps toward phasing out colistin in veterinary medicine and food-producing animals, international experience indicates that co-resistance to colistin and other critical agents may persist in some settings after formal restrictions, driven by complex ecological and co-selection dynamics (28). Within arid conservation facilities, high reliance on chemical disinfectants, challenges in fully removing organic material, and accumulation of residues in water systems and soils create conditions in which *qac*- and metal-tolerance genes could confer a fitness advantage (34). This context underscores the need to scrutinize not only antimicrobial use but also disinfection and environmental management practices when designing AMR mitigation strategies in wildlife centers.

The virulence gene complement of FAZ18016 is notable for its breadth and integrity. FAZ18016 retained full SPI-1 and SPI-2 islands, as well as T6SS loci and a broad effector spectrum that collectively support efficient intestinal invasion, intracellular survival and systemic dissemination (26). Comparable or even higher virulence gene loads have been reported in *Salmonella* isolates from intensively reared poultry and wild birds, highlighting that such virulence architectures can facilitate both disease and environmental persistence at the wildlife–livestock interface (17, 35). The extensive virulome of FAZ18016, combined with its MDR profile, suggests that ST198 lineages circulating in the UAE broiler chain remain fully capable of causing severe systemic disease in non-target hosts such as bustards, particularly under stress or other predisposing factors.

Phylogenomics indicated that FAZ18016 is closely related to *S*. Kentucky ST198 isolates that has been reported along UAE broiler chains (17, 18). The closest broiler isolates differed from FAZ18016 by only 31–33 SNPs, a distance comparable to that often observed within single outbreak clusters or transmission chains in bacterial genomic epidemiology (36). While the available dataset is limited and does not permit confident directionality inference, the simplest explanation is that FAZ18016 and these broiler isolates share a relatively recent common ancestor within local poultry-associated reservoirs. Potential pathways linking poultry operations and captive houbara include dust and aerosols in arid, windy environments; and synanthropic birds or rodents that move between farms and conservation centers (9–11). Although the case presented in our study cannot pinpoint the exact route of introduction, the genomic evidence supports the view that houbara breeding programs do not operate in isolation from surrounding livestock sectors and spillovers from poultry industry to houbara breeding need to be explicitly integrated into regional AMR surveillance and biosecurity planning.

This study has some limitations. It describes a single clinical case and cannot estimate the prevalence of *S*. Kentucky or *mcr*-1.1–positive strains in the local captive houbara populations; a goal that we will be investigating in the near future. Phylogenetic comparisons were limited to available poultry-derived isolates and did not include contemporaneous environmental or feed isolates from the houbara facility, precluding definitive source attribution. The nine genomes included represent the currently available published UAE genomes of *Salmonella* Kentucky. Although *S*. Kentucky ST198 is recognized globally as an important public health serovar, currently available UAE poultry genomic data indicates that other serovars, particularly *S*. Minnesota and *S*. Infantis, are more prevalent locally. Longitudinal surveillance would be required to determine whether similar strains persist or re-emerge within the conservation center. It is recommended that future case investigations should include broader sampling of non-fatal cases, healthy birds, and associated food and environmental sources to minimize selection bias and better assess transmission pathways. Clear reporting of any additional *Salmonella* isolates recovered, including those not selected for sequencing, would further strengthen epidemiological interpretation. Despite these limitations, the study's strengths remains the definitive genomic characterization of a clinically significant MDR *S*. Kentucky ST198 isolate from a threatened species, detailed reconstruction of the *mcr*-1.1–bearing plasmid, and demonstration of its close relatedness to local poultry strains, underscoring the value of integrated genomic approaches to reveal AMR connectivity between poultry production and wildlife conservation in Middle East contexts.

## 5 Conclusion

We report a yolk sac infection with poor prognosis in a captive Asian houbara bustard caused by an MDR *S. enterica* serovar Kentucky ST198 carrying *mcr*-1.1 on an IncHI2 plasmid co-harboring *tet(A), terC* and biocide tolerance genes. The isolate exhibited resistance to 17 antimicrobials and carried 25 AMR determinants and 156 virulence-associated genes. Phylogenomics demonstrated that the houbara isolate is closely related to broiler-associated ST198 strains from the UAE, differing by only a few dozen SNPs. These findings highlight how threatened wildlife in conservation programs can be exposed to and infected by high-risk MDR *Salmonella* lineages likely originating from poultry production systems. Plasmid-mediated colistin resistance, once established in broad-host-range IncHI2 backbones, may persist under co-selection by other antimicrobials, disinfectants and metals even after usage restrictions (32, 33). These findings highlight the need for expanded surveillance and for generating additional *Salmonella* genomes from wildlife, poultry, and related environmental sources with richer epidemiological metadata to better resolve transmission pathways and strengthen interpretation of One Health linkages in future studies.

## Data Availability

The sequencing data and assembled genomes generated in this study have been publicly deposited in the NCBI database under BioProject accession number PRJNA1354649.
